# A state-level study of opioid use disorder treatment access and neonatal abstinence syndrome

**DOI:** 10.1186/s12887-019-1718-x

**Published:** 2019-10-23

**Authors:** Elizabeth R. Wolf, Sebastian T. Tong, Roy T. Sabo, Steven H. Woolf, Kassie Abbinanti, James Pecsok, Alex H. Krist

**Affiliations:** 1 Department of Pediatrics, Virginia Commonwealth University, 1000 East Broad Street, Richmond, VA 23219 USA; 2 Children’s Hospital of Richmond at VCU, Richmond, VA USA; 3 Department of Family Medicine and Population Health, Virginia Commonwealth University, Richmond, VA USA; 4 Department of Biostatistics, Virginia Commonwealth University, Richmond, VA USA; 5 Center on Society and Health, Richmond, VA USA

**Keywords:** Neonatal, Abstinence, Opioid, Substance, Withdrawal

## Abstract

**Background:**

Adult opioid use and neonatal abstinence syndrome (NAS) are growing public health problems in the United States (U.S.). Our objective was to determine how opioid use disorder treatment access impacts the relationship between adult opioid use and NAS.

**Methods:**

We conducted a cross-sectional state-level ecologic study using 36 states with available Healthcare Cost and Utilization Project State Inpatient Databases in 2014. Opioid use disorder treatment access was determined by the: 1) proportion of people needing but not receiving substance use treatment, 2) density of buprenorphine-waivered physicians, and 3) proportion of individuals in outpatient treatment programs (OTPs). The incidence of NAS was defined as ICD-9 code 779.5 (drug withdrawal syndrome in newborn) from any discharge diagnosis field per 1000 live births in that state.

**Results:**

Unmet need for substance use disorder treatment correlated with NAS (r = 0.54, 95% CI: 0.26–0.73). The correlation between adult illicit drug use/dependence and NAS was higher in states with a lower density of buprenorphine-waivered physicians and individuals in OTPs.

**Conclusions:**

Measures of opioid use disorder treatment access dampened the correlation between illicit drug use/dependence and NAS. Future studies using community- or individual-level data may be better poised to answer the question of whether or not opioid use disorder treatment access improves NAS relative to adult opioid use.

## Background

Neonatal abstinence syndrome (NAS) is a drug withdrawal syndrome experienced by newborns whose mothers have taken opioids or other substances during pregnancy. Opioids that can cause NAS include illicit substances (e.g., heroin) as well as legally prescribed medications (e.g., oxycodone, methadone and buprenorphine). It is estimated that 60–80% of infants exposed to opioids develop NAS [1]. Clinical manifestations of acute NAS include irritability, hypertonicity, jitteriness, diarrhea, and failure to thrive [2]. Moderate to severe NAS (27–91% of cases) requires treatment with opioids such as morphine and methadone [2]. The need for additional monitoring and treatment of NAS results in prolonged hospital stays and high medical costs [2].

Concurrent with the surge in adult opioid use across the United States [3], the incidence of NAS has also increased dramatically—from 1.5 per 1000 hospital births in 1999 to 14.4 per 1000 in 2014 [4]. The incidence of NAS varies more than 30-fold between states; ranging from 0.7 cases per 1000 live births in Hawaii to 33.4 per 1000 in West Virginia [5]. There are two primary long-acting oral opioids that are used for adult opioid use disorder treatment: methadone and buprenorphine. Methadone is typically given in observed clinical settings, whereas buprenorphine is typically taken by patients in their own homes. There is growing use of buprenorphine in pregnant women as infants born to women taking buprenorphine have better outcomes (e.g.: length of stay, birth weight) compared with infants born to women taking methadone [6]. Since the medicines that are used for *treating* opioid use disorder in pregnant women (e.g. methadone and buprenorphine) can also *result in* NAS, [7, 8] the relationship between opioid use disorder treatment access and NAS is not yet well understood. Furthermore, it has been difficult to compare rates of NAS between infants of mothers using illicit drugs such as heroin and those using prescribed medications for opioid use disorder such as methadone and buprenorphine. Randomized controlled trials on this topic are considered unethical and it is challenging to control for factors that might influence a pregnant woman’s enrollment in an opioid use disorder treatment program within observational studies.

In addition to opioid replacement therapy, opioid use disorder treatment programs may also include prenatal care, case management, behavioral and mental health interventions and social service support. We hypothesized that there may be non-pharmacologic ways in which opioid use disorder treatment programs might lower rates of NAS such as improved mother-infant bonding, increased breastfeeding, and decreased use of non-opioid drugs [9]. This state-level analysis examined whether NAS was associated with 1) the proportion of individuals needing but not receiving substance use disorder treatment (unmet need), 2) the density of buprenorphine-waivered physicians, and 3) the number of individuals in outpatient treatment programs (OTPs) per 100,000 population. We hypothesized that: 1) measures of adult opioid use (illicit drug use/dependence and opioid prescribing) would be positively correlated with NAS, and 2) better opioid use disorder treatment access would dampen the positive association between adult opioid use and NAS.

## Methods

### Study design

We conducted a cross-sectional state-level ecologic analysis of pediatric discharge data from the Healthcare Cost and Utilization Project’s State Inpatient Databases (HCUP SID) and state-level variables from other publicly-available databases.

### Data sources, exposures and covariates

The percentage of patients needing but not receiving treatment was obtained from open publicly available databases including the National Survey on Drug Use and Health (NSDUH), which provides state-level data on illicit drug dependence or abuse in the past year and individuals who needed but did not receive treatment for substance use [10]. The extent of the unmet substance use treatment need in the United States is measured by calculating the number of people aged 12 or older who were classified as having substance use disorder based on the NSDUH, but who did not receive substance use treatment at a specialty facility in the past year [11]. State data on the number of individuals enrolled in OTPs were obtained from the Substance Abuse and Mental Health Services Administration [12]. State data on the number of buprenorphine-waivered physicians were gathered from the Drug Enforcement Administration (DEA) database [13]. The density (number per 100,000 population) of buprenorphine-waivered physicians was considered a proxy for opioid use disorder treatment access since a DEA waiver is required to prescribe buprenorphine, one of the two primary medications used to treat opioid use disorder. While some buprenorphine-waivered physicians may not provide care for pregnant women, these measures reflect general access to opioid use disorder services. We used the Intercontinental Marketing Services Health National Prescription Audit database to obtain physician prescribing rates for opioids [14]. State-level data on demographic characteristics (percentage of Hispanic or African American residents) and the proportion of the population living in poverty or in rural areas was gathered from the U.S. Census Bureau [15]. All exposure and covariates were gathered from the most recent year available.

### Outcome

We identified cases of NAS by abstracting ICD-9 code 779.5 (drug withdrawal syndrome in newborn) [1] from any discharge diagnosis field within the most recent HCUP SID for pediatric hospitalizations [16]. Because this time frame used ICD-9 rather than ICD-10 codes, we were unable to distinguish between infants who experienced withdrawal from illicit opioids and those who experienced withdrawal from prescribed opioids. We identified 35 states that had publicly available data on NAS through the centralized HCUP SID from 2014. The Virginia Department of Health also provided NAS SID discharge data from 2014 [17] making a total of 36 states with available outcome data. The incidence of NAS was reported as the number of NAS cases per 1000 live births in each state in 2014 [18].

### Data analysis

Associations between continuous measurements and NAS were measured using Pearson linear correlation coefficients, while NAS was compared between levels of binary classifications using an equal variance two-sample t-test and was compared between levels of polytomous classifications using the Kruskal-Wallis test (due to small group sizes). For any prevalence measures that had significant associations with NAS, analysis of covariance without interaction was used to reassess that association for significance (and sign: positive or negative) in the presence of access and demographic measurements. Due to small sample size, each access/demographic measurement was included separately, and no interaction effects were modeled. The study’s relatively small sample size (*n* = 36) prevented an analysis of opioid use disorder treatment access measures as classical effect modifiers (i.e. interaction terms). Instead, states were stratified as having low or high density of buprenorphine-waivered physicians and individuals in OTPs, based on whether they were above or below the national median. The MEANS, CORR and GLIMMIX procedures in the SAS Statistical Software platform (version 9.4, Cary, NC, USA) were used for all summaries and analyses. NAS frequencies were mapped using EsriPress ArcGIS 10.2.

## Results

### Variability of access and NAS

**Fig. 1:**
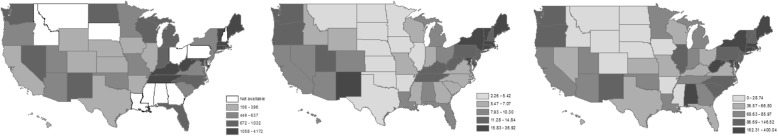
Incidence of neonatal abstinence syndrome per 1000 live births, buprenorphine-waivered physicians per 100,000 population and individuals in outpatient treatment programs per 100,000 population, by state. Figure was generated by authors

Although the median density of buprenorphine-waivered physicians was 8 (IQR: 5–14) per 100,000 population (Table 1), the concentrations varied substantially across the states, ranging from 2 per 100,000 in Iowa to 37 per 100,000 in Vermont (Fig. 1). Similarly, although the median OTP caseload was 80 (IQR: 46–114) individuals per 100,000 population, the values ranged from zero in North Dakota to 278 per 100,000 in Maryland (Table 1).

**Table 1:** Summaries of opioid use, access, demographic and outcome measurements

Opioid Use	Median (IQR)
Illicit drug dependence or abuse per 1000 population	22 (20–25)
Opioid prescribing rates per 1000 population	774 (712–948)
Access	
Number of buprenorphine -waivered physicians per 100,000 population	8 (5–14)
Number of individuals in outpatient treatment programs per 100,000 population	80 (46–114)
Unmet need (needing but not receiving treatment for illicit drug use) per 1000 population	20 (18–21)
Demographics	
Proportion of state population with incomes below the federal poverty level	13% (11–16%)
Proportion of state population with rural domicile	25% (11–34%)
Proportion of state population that is Hispanic	10% (5–17%)
Proportion of state population that is African American	6% (3–14%)
Outcome	
Number of NAS cases per 1000 hospital discharges under age 1	6 (4–10)

The median incidence of NAS was 6 per 1000 live births, ranging from 1 per 1000 live births in Hawaii to 36 per 1000 live births in Vermont. As shown in Table 2, incidence rates of NAS correlated with state rates for illicit drug dependence or abuse and opioid prescribing (r = 0.36, 0.36, respectively; Table 2). NAS was more common in more rural states and less common in states with higher proportions of Hispanics (Table 2). NAS was not significantly associated with the proportion of African-Americans or those living in poverty (Table 2).

**Table 2:** Correlations between neonatal abstinence syndrome and other predictive factors

	Correlation (r; 95% CI)	*p*-value
Adult opioid use		
Proportion of individuals with illicit drug dependence or abuse	0.36 (0.04–0.61)	0.03
Rate of opioid prescribing	0.36 (0.05–0.61)	0.02
Access		
Number of buprenorphine-waivered physicians per 100,000 population	0.71 (0.50–0.84)	< 0.001
Number of individuals in outpatient treatment programs per 100,000 population	0.54 (0.26–0.73)	< 0.001
Demographics		
Proportion of state population with incomes below the federal poverty level	0.13 (−0.20–0.43)	0.44
Proportion of state population with rural domicile	0.61 (0.35–0.77)	< 0.001
Proportion of state population that is Hispanic	− 0.38 (− 0.62 - -0.07)	0.02
Proportion of state population that is African American	− 0.14 (− 0.44–0.19)	0.41

### Treatment access and NAS

NAS correlated with levels of unmet need for substance use disorder treatment (r = 0.54, 95% CI: 0.26–0.73). The correlation between NAS and illicit drug use/dependence was stronger in states with a *lower* density of buprenorphine-waivered physicians and *lower* proportion of individuals in OTPs (Table 3). In contrast, the correlation between NAS and opioid prescribing was stronger in states with *higher*-density of buprenorphine-waivered physicians (Table 3).

**Table 3:** Correlations between neonatal abstinence syndrome and measures of adult opioid use stratified by density of buprenorphine-waivered physicians and outpatient treatment program client case load

	Correlation (r; 95% CI)	*p*-value
Above median number of buprenorphine waivered physicians per 100,000 population (*n* = 20)^1^		
Proportion of individuals with illicit drug dependence or abuse	−0.04 (− 0.48–0.41)	0.86
Rate of opioid prescribing	0.48 (0.03–0.75)	0.03
Below median number of buprenorphine waivered physicians per 100,000 population (*n* = 18)^1^		
Proportion of individuals with illicit drug dependence or abuse	0.73 (0.38–0.88)	< 0.001
Rate of opioid prescribing	0.39 (−0.10–0.72)	0.12
Above median number of clients in outpatient treatment program per 100,000 population (*n* = 20)^1^		
Proportion of individuals with illicit drug dependence or abuse	0.33 (−0.14–0.67)	0.16
Rate of opioid prescribing	0.27 (−0.21–0.63)	0.26
Below median number of clients in outpatient treatment program per 100,000 population (*n* = 18)^1^		
Proportion of individuals with illicit drug dependence or abuse	0.59 (0.15–0.82)	0.01
Rate of opioid prescribing	0.38 (−0.11–0.72)	0.12

^1^“High” is above whereas “low” is below the national median; where the national median density of buprenorphine-waivered physicians = 8 per 100,000 population and the national median density of clients in outpatient treatment programs = 75 per 100,000 population

## Discussion

### Patterns of neonatal abstinence syndrome

As expected, we found that higher state averages for adult opioid prescribing and illicit drug use correlated with higher rates of NAS. Adult opioid use and NAS have both dramatically increased across the U.S. over the past 10 years [3, 5]. The geographic and demographic trends of NAS that we observed are similar to those observed with adult opioid use. For example, states with the highest NAS incidence—Maine, Vermont, West Virginia and Tennessee—are also known to have major adult opioid crises [3]. Similarly, in our study, NAS was more common in states with a higher proportion of people living in rural domiciles. This is consistent with a recent national study that found NAS to be more prevalent in rural counties [19]. We also found that NAS was less common in states with greater proportions of Hispanics. This is consistent with data demonstrating that the largest increases in prescription-overdose deaths and heroin-overdose deaths are amongst whites [20]. Other studies have found that white patients tend to have greater access to opioid prescriptions in emergency rooms [21] and that opioid prescriptions are more common in white pregnant women compared with pregnant women of other races [7]. Interestingly, the state’s proportion living in poverty did not seem to correlate with NAS perhaps because of our limited sample size, the lack of individual-level data or the interaction between race and poverty at the state level.

### Patterns of substance use disorder treatment access

Between states, there was wide variation in access to opioid use disorder treatment. The states with the highest levels of access tended to be in the Northeast and the Pacific Northwest. New Mexico also had high levels of opioid use disorder treatment access (Fig. 1). The Appalachian states (Tennessee, Kentucky and West Virginia) had some of the highest NAS incidence rates but had variable levels of access to opioid use disorder treatment. Kentucky had a low proportion of individuals in OTPs, whereas West Virginia had a high proportion of individuals in OTPs. All three had moderate levels of buprenorphine-waivered physicians (Fig. 1).

### Relationship between treatment access and neonatal abstinence syndrome

**Fig. 2:**
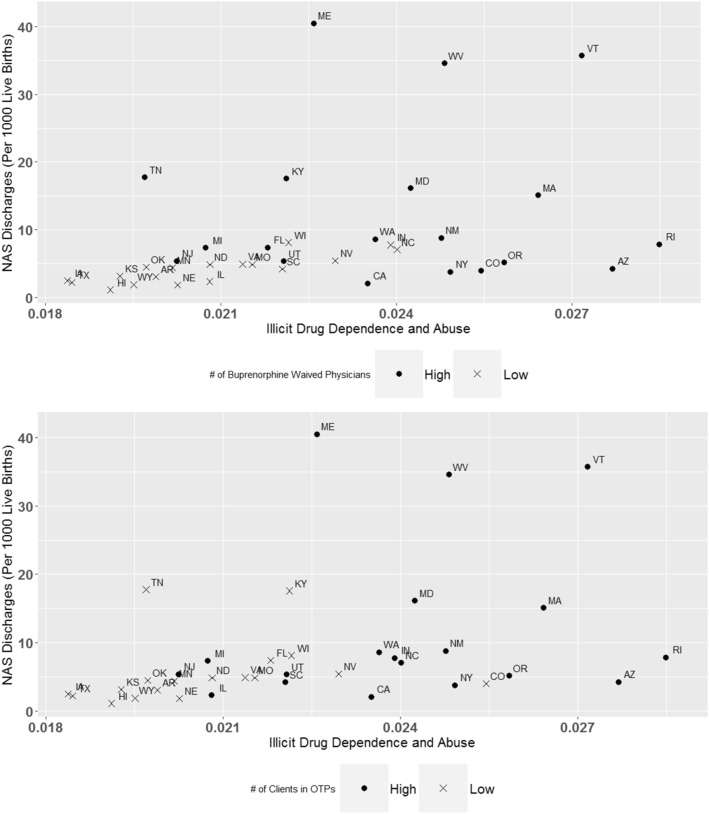
Cases of neonatal abstinence syndrome vs. illicit drug dependence and abuse, stratified by the density of buprenorphine-waivered physicians and individuals in outpatient treatment programs. “High” and “Low” densities refer to a density of buprenorphine-waivered physicians and individuals in outpatient treatment programs above and below the national median (8 physicians per 100,000 population and 80 individuals per 100,000 population, respectively)

Unmet need for substance use disorder treatment was positively and significantly correlated with NAS. This finding is in concordance with a county-level study that found an association between shortage of mental health providers and NAS [22]. However, the role of buprenorphine-waivered physicians and OTPs on the relationship between adult opioid use and NAS was less clear. There was a significant correlation between illicit drug use/dependence and NAS in states with lower levels of opioid use disorder treatment access (both density of buprenorphine-waivered physicians and individuals in OTPs). In states with higher levels of treatment access, the correlation between NAS and illicit drug use/dependence became non-significant. These findings were largely driven by the wide variability in NAS in high-access states (Fig. 2). Figure 2 shows that in particular, Maine, West Virginia and Vermont had very high NAS rates relative to rates of illicit drug use/dependence. One explanation for these outliers is that there may be differences in the way NAS is recognized or recorded in these states [23]. To address this problem, some states are moving towards creating NAS registries. However, these are not widespread enough to study trends across states. Another explanation for these outlying states is that there was increased use of non-opioid drugs that increased NAS relative to illicit drug use.

In contrast to illicit drug use/dependence, the correlation between NAS and opioid prescribing was actually stronger in states with a *greater* density of buprenorphine-waivered physicians. One explanation for this phenomenon is that buprenorphine itself can result in NAS. There may also be a diversion of prescribed buprenorphine to unintended recipients. In addition, some states have graduate medical education that includes buprenorphine-waivers for residents, but some of these physicians may not actually use the waivers for opioid use disorder treatment.

### Potential mechanisms

There are several potential mechanisms for how access to opioid use disorder treatment might reduce the likelihood of NAS. First, long-acting opioids used in opioid use disorder treatment may pose a lower risk for NAS than do short-acting drugs such as heroin or oxycodone [24] due to steadier-state concentrations in the blood. As mentioned previously, this hypothesis is difficult to test in randomized controlled trials because it is considered unethical to not treat pregnant women with opioid use disorders. Observational studies have shown that the proportion of infants with NAS born to pregnant women taking heroin (40–80%) overlap those treated with methadone (13–94%) and buprenorphine (22–67%) [2]. Second, women taking methadone or buprenorphine are often permitted to breastfeed whereas women using heroin women are not. Breastfeeding may reduce NAS through opioid transfer in breastmilk and through non-pharmacologic means through physical proximity and comforting [25]. Third, opioid use disorder treatment programs tend to have lower rates of non-opioid drug use, which may further reduce the likelihood of NAS [26, 27]. Fourth, opioid use disorder treatment programs may include parenting, behavioral health or mental health interventions that can improve mother-infant bonding [28, 29]. Fifth, opioid use disorder treatment may reduce NAS by non-pharmacologic physiologic mechanisms, such as lowering maternal cortisol levels [30].

### Limitations

Our study has several limitations. The first limitation is missing data: we lacked NAS data for 14 (28%) of the 50 states. Lacking a national database for NAS, we relied on data from individual states, some of which do not participate in centralized HCUP distribution. We do not know if exclusion of these 14 states biased our results and what direction that bias may have taken. Second, the study relied on ICD-9 codes for diagnoses of NAS. ICD-9 codes have been used widely by groups such as the Centers for Disease Control and Prevention to characterize NAS trends. Nevertheless, ICD-9 codes have several limitations. ICD-9 codes can be generated by non-medically trained personnel and may not always accurately reflect patients’ medical conditions. Some studies have found that ICD-9 codes tend to underestimate cases of NAS [31, 32]. We would expect this type of bias to be non-differential and not affect our comparisons between states. Another problem with ICD-9 codes is that unlike the ICD-10 system (instituted in 2015), ICD-9 codes cannot distinguish between withdrawal from illicit opioids and iatrogenic or prescribed opioids. Furthermore, ICD-9 codes do not distinguish between withdrawal from opioids and non-opioid drugs. If there were differences in non-opioid drug use between states, these may have affected our results. When more recent data becomes available, we may be able to study this topic with more precision through use of ICD-10 codes such as P96.1 (neonatal withdrawal symptoms from maternal use of drugs of opioid use disorder) and P96.2 (withdrawal symptoms from therapeutic use of drugs in newborn). Furthermore, since the HCUP SID includes discharge diagnoses from multiple hospitals, if an infant is transferred from one hospital to another, that infant may be counted more than once. This phenomenon may be more likely to occur in rural locations.

Our small sample size and inability to control for other state-level factors did not allow us to identify other characteristics that could have moderated the effect of adult opioid use on NAS. We did not have data on how many pregnant women had opioid use disorder in various states. The proportion of buprenorphine-waivered physicians and individuals in OTPs are indirect measures of treatment access. Certain clinics may have improved access for pregnant women relative to men and non-pregnant women. Other factors that could conceivably influence the relationship between adult opioid use and NAS include 1) the fertility rate amongst opioid-addicted mothers, 2) the proportion of pregnant women using non-opioid drugs such as tobacco, anti-depressants or benzodiazepines (all of which have been shown to increase rates of NAS) 3) the comprehensiveness or content of opioid use disorder-treatment programs in each state, 4) the promotion of abstinence vs. opioid replacement within opioid use disorder treatment programs, 5) the ratio of public to private treatment programs 6) the willingness of licensed providers to treat pregnant women and 7) variability in the legal measures taken against substance-abusing mothers [7, 33]. Some opioid use disorder programs include behavioral interventions, mental health treatment and social service support. These more comprehensive programs may be more successful in reducing rates of NAS. Future studies could examine the relationship between the components of substance use disorder treatment programs and incidence of NAS. States can have different interpretations of the Child Abuse Prevention and Treatment Act (CAPTA), which requires notification of child protective services for children affected by drug withdrawal [34–36]. It is possible that in more punitive states, pregnant women may be less likely to seek opioid use disorder treatment even if it were accessible to them.

In addition, our findings—which rely on state-level statistics—are vulnerable to the “ecologic fallacy,” in which inferences drawn from a population do not apply to individual members. For example, just because a state has a certain proportion of physicians who can prescribe buprenorphine, does not mean that these physicians are equally distributed throughout the population or accessible to those in need. Furthermore, there is a wide variability in how many patients each buprenorphine-waivered physician treats [37]. Future studies using community- or individual-level data may be better poised to answer the question of whether or not opioid use disorder treatment access improves NAS relative to adult opioid use. Lastly, our cross-sectional design did not allow for an analysis of temporal relationships. We do not think this is a major concern with our study, however, since we are studying exposures that affect adults, specifically mothers, (who are by definition born before infants) and an outcome that affects only infants.

## Conclusions

In summary, unmet need for substance use disorder treatment was positively correlated with NAS. The correlation between adult illicit drug use/dependence and NAS was dampened in states with higher levels of opioid use disorder treatment access. Future studies utilizing community or individual level data may be better able to address whether adding opioid use disorder treatment programs or buprenorphine-waivered physicians can reduce incidence of NAS. Since NAS results in substantial infant morbidity and healthcare costs, the impact of NAS should be taken into account when considering public health measures that would improve access to opioid use disorder treatment.

## Data Availability

The data for this project are publicly available and relevant links can be found in the references section.

## Abbreviations

CAPTA: Child Abuse Prevention and Treatment Act
DEA: Drug Enforcement Administration
HCUP SID: Healthcare Cost and Utilization Project’s State Inpatient Databases
NAS: Neonatal Abstinence Syndrome
NSDUH: National Survey on Drug Use and Health
OTPs: Outpatient treatment programs
